# Population-based ultrasound reference values for inter-rectus distance: threshold prevalence, percentiles, and the limitations of fixed cutoffs

**DOI:** 10.1007/s10029-026-03810-8

**Published:** 2026-08-31

**Authors:** Annukka Marttila, Katariina Kilpivaara, Jenni Kinnunen, Susanna M Savukoski, Pasi P Ohtonen, Riikka K Arffman, Lotta Vuokila, Marika H Kangasniemi, Kaisu Luiro, Maria Rajecki, Tero Rautio, Terhi T Piltonen

**Affiliations:** 1 Kainuu Central Hospital, Kajaani, Finland; 2 Translational Medicine Research Unit, University of Oulu, Oulu, Finland; 3 Oulu University Hospital, Oulu, Finland; 4 Department of Obstetrics and Gynecology, Oulu University Hospital, Oulu, Finland; 5 Research Unit of Clinical Medicine, Medical Research Centre, University of Oulu, Oulu, Finland; 6 Research Service Unit, Oulu University Hospital, Oulu , Finland; 7 Department of Obstetrics and Gynecology, Reproductive MedicineUnit, Helsinki University Hospital, Helsinki, Finland; 8 University of Helsinki, Helsinki, Finland

**Keywords:** Diastasis recti, weakness of the linea alba, abdominal muscles, rectus abdominis, birth cohort

## Abstract

**Purpose:**

This population-based study aimed to characterize the distribution of inter-rectus distance (IRD) and its upper range in adult women using standardized multisite ultrasound measurements and a percentile-based interpretive framework.

**Methods:**

Data was driven from the Finnish Women’s Health Study (WENDY, 2020–2023), comprising women assigned female at birth with a mean age of 35 years (age range 33–37 years, SD 0,6). Women less than six months postpartum, exclusively breastfeeding, or with prior midline abdominal surgery or hernia repair involving a midline incision or abdominoplasty were excluded. Inter-recti distance was measured at six predefined locations along the linea alba in the supine, knee-flexed resting position using ultrasound. For the analysis, the maximal IRD of each examinee across all sites was used.

**Results:**

A total of 1,840 women with naturally occurring anatomy were examined. At the standard site 3 cm above the umbilicus, the mean IRD was 2.37 cm (SD 1.19 cm; range 0.35–9.86 cm). Nulliparous women had a lower mean IRD (1.52 cm, SD 0.70 cm; 0.35–4.37 cm, *n* = 539) than parous women (2.71 cm, SD 1.18 cm; 0.46–9.86 cm, *n* = 1,301). The cohort’s 80th percentile for IRD was 3.24 cm. Based on maximal IRD, 38.5% had IRD ≥ 3 cm and 5.4% exceeded 5 cm; the 95th percentile was 5.09 cm. Maximal IRD most often occurred at the level of the umbilicus (41.3%) or 3 cm above (44.1%) and rarely below the umbilicus. Maximal IRD correlated weakly with adiposity measures (body mass index (BMI) ρ = 0.227; waist ρ = 0.273; weight ρ = 0.199) and no correlation was observed with height. Regression analysis showed that each childbirth was associated with a 0.42 cm increase in maximal IRD, and BMI increased IRD by 0.061 cm per kg/m².

**Conclusion:**

IRD is a continuous anatomical characteristic exhibiting considerable normal variation among adult women. A maximal IRD of up to 5 cm may be regarded as being within the upper tail of the population reference distribution. Fixed cutoff values should be proportionate to clinical symptoms, in order to prevent unnecessary interventions. Altogether, our findings provide population-based reference data for interpreting IRD measurements.

## Introduction

The inter-rectus distance (IRD) quantifies the separation between the medial borders of the rectus abdominis muscles and reflects the width of the linea alba, a fibrous midline structure formed by interweaving aponeuroses of the lateral abdominal wall muscles. Anatomical and morphometric studies have demonstrated that the width of the linea alba varies continuously along its cranio-caudal axis and between individuals, without distinct biological thresholds [1–3]. Radiological evidence further supports the interpretation of normal abdominal wall anatomy as a spectrum of variations best characterized at the population level rather than by fixed numerical cutoffs [4, 5].

IRD is a measurement; clinically significant diastasis recti abdominis (DRA) is a broader construct that may require symptoms, functional impairment, bulging, patient-reported consequences, or treatment context. Despite this, clinical literature often merges IRD with DRA, a structural concept typically defined by fixed cutoffs. This type of categorization overlooks the continuous and right-skewed distribution of IRD values in adult populations and may lead to misclassification of normal anatomical variations as pathological [6, 7]. Existing research has focused frequently on pregnancy-related or early postpartum populations or narrowly selected groups, such as nulliparous women, limiting the understanding of IRD variations in the general adult female population [8, 9].

Interpretation of IRD is further complicated by substantial methodological heterogeneity. The assessment of IRD varies depending on the measurement modality—whether through it is clinical palpation, ultrasound, computed tomography (CT)—as well as the anatomical measurement site, body position, and respiratory phase. These factors contribute to considerable variation in reported IRD values and hinder comparability between studies [10–12]. Notably, the IRD magnitude depends significantly on the measurement location along the linea alba, with values typically being the widest supraumbilically and narrowing caudally [8, 13, 14].

Population-based imaging studies illustrate these limitations of fixed cutoff approaches. For example, Kaufmann et al. reported that 57% of adults exceeded the commonly used 3 cm above umbilicus, despite IRD values forming a continuous, right-skewed distribution without a distinct abnormal subgroup [13]. Similar high-resolution morphometric analyses have confirmed a gradual, location-dependent variation in natural anatomical thresholds of the linea alba width [15].

Overall, these findings highlight the lack of established population-based reference frameworks for IRD interpretation in adult females. Such frameworks should be percentile-based to capture continuous anatomical variations across multiple measurement sites, independent of pregnancy or postpartum status. This approach enables the differentiation between common anatomical variations and rare extreme values, providing a coherent basis for both clinical decision-making and research applications.

The aim of this study was to characterize the population-based distribution of IRD in women using standardized ultrasound measurements at multiple predefined anatomical sites. By defining the IRD as the maximal separation observed along the linea alba in each examinee, this study aimed to describe the statistic normal anatomical variation and its upper range within a representative adult female cohort.

## Materials and methods

### Study design and population

This study used data from the Women’s Health Study (WENDY), a population-based cohort study conducted in Finland between 2020 and 2023 [16]. The WENDY study included *n* = 1,916 women (assigned female at birth, AFAB) aged approximately 35 years at baseline. All examinees in the study were recruited from Finland. Although data on self-reported ethnicity were gathered, they were not analyzed in this part of the research.

**Fig. 1:**
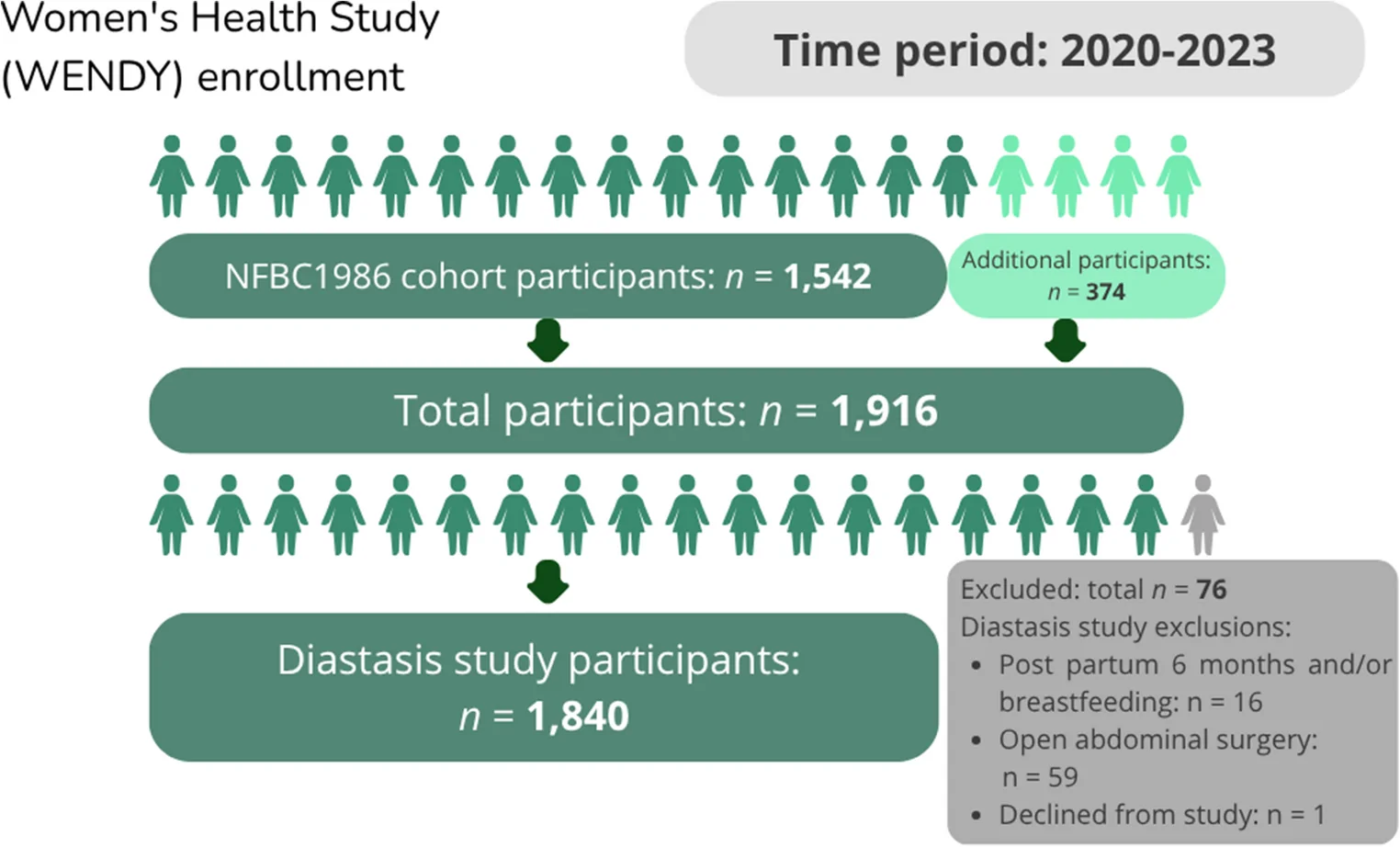
Flowchart of the WENDY study population having ultrasonographically measured inter-rectus distance

Female members of the Northern Finland Birth Cohort 1986 with a registered address in Finland on January 1, 2020, were invited to participate in the study [17–19]. In addition, the cohort was supplemented with *n* = 1,112 randomly selected women born in the same geographical area up to 1.5 years after the last birth in the NFBC1986 cohort, of whom *n* = 374 participated. Study visits were conducted at the Oulu and Helsinki study sites between 2020 and 2023. All participants provided written informed consent. The study flowchart is presented in Fig. 1.

### Data collection and exclusion criteria

Participants completed an extensive study visit to screen for gynecological, anatomical and metabolic factors affecting women’s health, including IRD measured with ultrasound. Basic anthropometric measures, such as weight and height, were also taken with scale and measuring tape. The examinees also completed a questionnaire, including questions about awareness of DRA and parity.

For the present analyses, women, who had delivered within the preceding six months (*n* = 11) or were exclusively breastfeeding at the time of the study visit, were excluded from the analysis. Participants with a history of open midline abdominal surgery, hernia repair involving a midline incision, or abdominoplasty were also excluded (total *n* = 59), as such procedures may alter the natural anatomy of the linea alba. One participant declined to undergo ultrasound measurements.

### IRD measurement

As an independent research component within WENDY, IRD was assessed using a standardized abdominal ultrasound following basic anthropometric measurements (height and weight). Ultrasonography was performed using a Samsung HS60 system with a linear probe. Measurements were performed by four gynecologists, specially trained in IRD measuring.

**Fig. 2:**
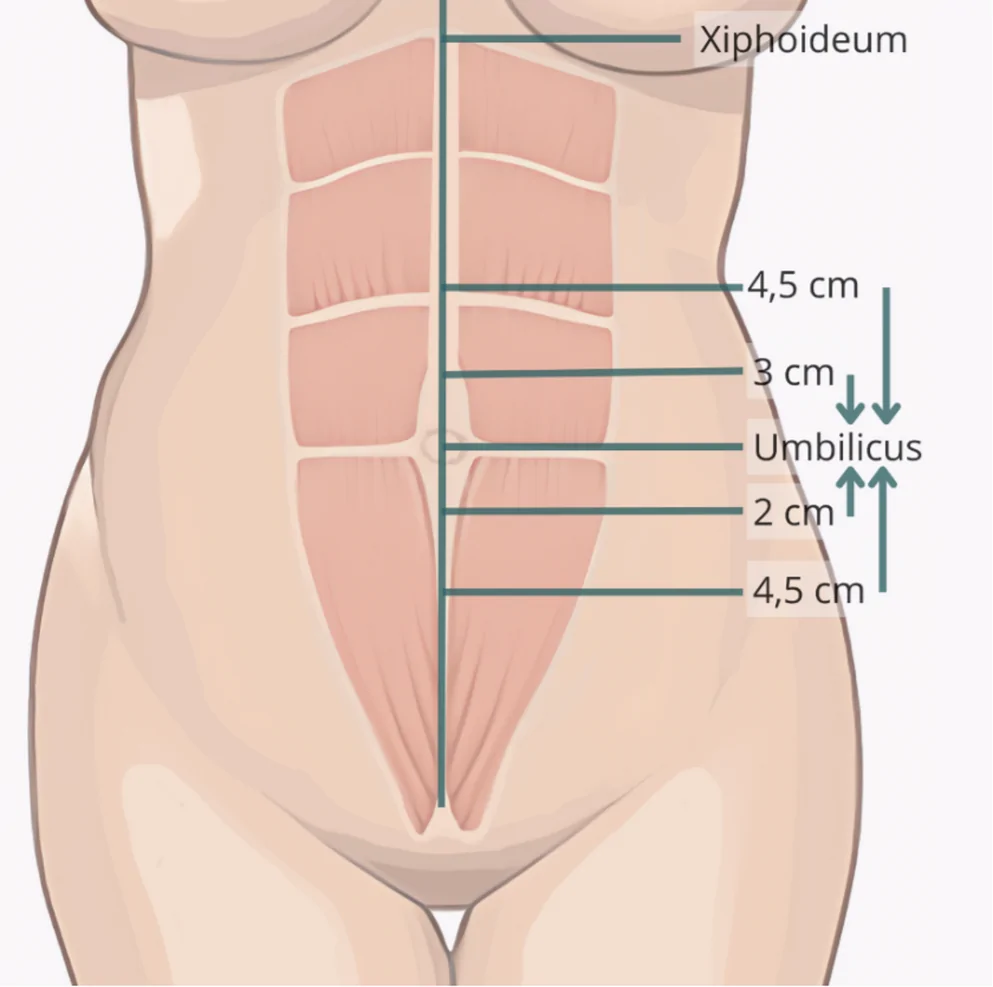
Six different measuring points of the inter-rectus distance in the women’s health study

The IRD was measured at predefined anatomical reference points along the linea alba. The primary measurement sites were those described by Beer et al., namely at the xiphoid, 3 cm above the umbilicus, and 2 cm below the umbilicus [8]. Additional measurement sites based on the classification proposed by Rett et al., umbilicus level, 4.5 cm above and 4.5 cm below the umbilicus, were included to capture broader spatial variations [20]. The measurement sites are illustrated in Fig. 2.

All measurements were performed with the participants in the supine position, knees slightly flexed, and abdominal muscles relaxed to minimize muscular activation.

### Definition of IRD for analyses

For analytical purposes, IRD was also defined as the maximal distance observed at any horizontally measured anatomical location along the linea alba. This approach was chosen to reflect the full spatial expression of abdominal midline separation rather than relying on a single, standardized measurement point.

### Statistical analysis

The mean and standard deviation (SD) or median with 25th -75th percentiles are presented as summary measures. Spearman’s correlation coefficient was calculated to present correlations of the different variables. SPSS for Windows (IBM Corp. Released 2023. IBM SPSS Statistics for Windows, Version 29.0.0 Armonk, NY: IBM Corp) was used for data analysis.

## Results

### Study population

The analyses of IRD were based on an analytical sample of 1,840 participants. The characteristics of the study population are presented in Table 1.

**Table 1:** Descriptive characteristics of the inter-rectus distance study population (*n* = 1,840). The number of participants varies due to absent data for certain variables

Anthropometric characteristics					
	*n*	Minimum	Maximum	Mean	Std. Deviation
Age	1840	33.9	37.2	35.3	0.59
Height (cm)	1839	145.9	187.0	165.3	5.92
Weight (kg)	1837	42.5	163.4	71.1	15.57
BMI (kg/m2)	1837	16.27	60.60	26.0	5.48
Total	1840				
Parity					
	*n*	Percent			
Nulliparous (0)	539	28.1			
Parity (1)	336	17.5			
Parity (2)	639	33.4			
Parity (≥ 3)	402	21			
	n	Minimum	Maximum	Mean	St. Deviation
Number of deliveries	1916	0	10	1.66	1.53
Other characteristics					
Ethnicity	*n*	Percent			
Caucasian*	1870	97.6			
Sami	43	2.2			
Unknown for examinee	3	0.2			
Total	1916	100			
Heard of diastasis recti abdominis					
	*n*	Percent			
No	142	7.4			
Yes	1449	75.6			
Limited understanding	325	17			
Total	1916	100			

*The term “Caucasian” reflects the original survey wording. It is recognized as an imprecise and outdated descriptor and should not be interpreted as equivalent to White, Western European or European race, ethnicity, nationality, or genetic ancestry

The participants were predominantly of “Caucasian”^1^ ethnicity by self-reportation. The median body mass index (BMI) was 26.02 kg/m² (range 16.25–60.68 kg/m²). Parity ranged from 0 to 10 (mean 1.66, median 2.0). Of the included participants, 539 (28.1%) were nulliparous, 336 (17.5%) had childbirth once, 639 (33.4%) twice, and 402 (20.1%) three or more times. While 76% (*n* = 1449) had heard of the DRA, only 7% (*n* = 142) had not, and 17% (*n* = 325) had a limited understanding of it (Table 1).

The majority of the participants (75.6%) reported having heard of the concept of diastasis recti abdominis prior to the examination (Table 1).

### Distribution of IRD distance at a standard measurement site

The mean maximal inter-rectus distance from any measuring point was 2.77 cm (SD 1.30), the median was 2.59 cm, the interquartile range was 1.85–3.47 cm, and the range was 0.39–12.63 cm. An IRD measured 3 cm above the umbilicus was used as the standard reference measurement. Among the participants included in the analyses (*n* = 1,840), the mean IRD at this location was 2.37 cm (SD 1.19), with values ranging from 0.35 to 9.86 cm (Table 2).

**Fig. 3:**
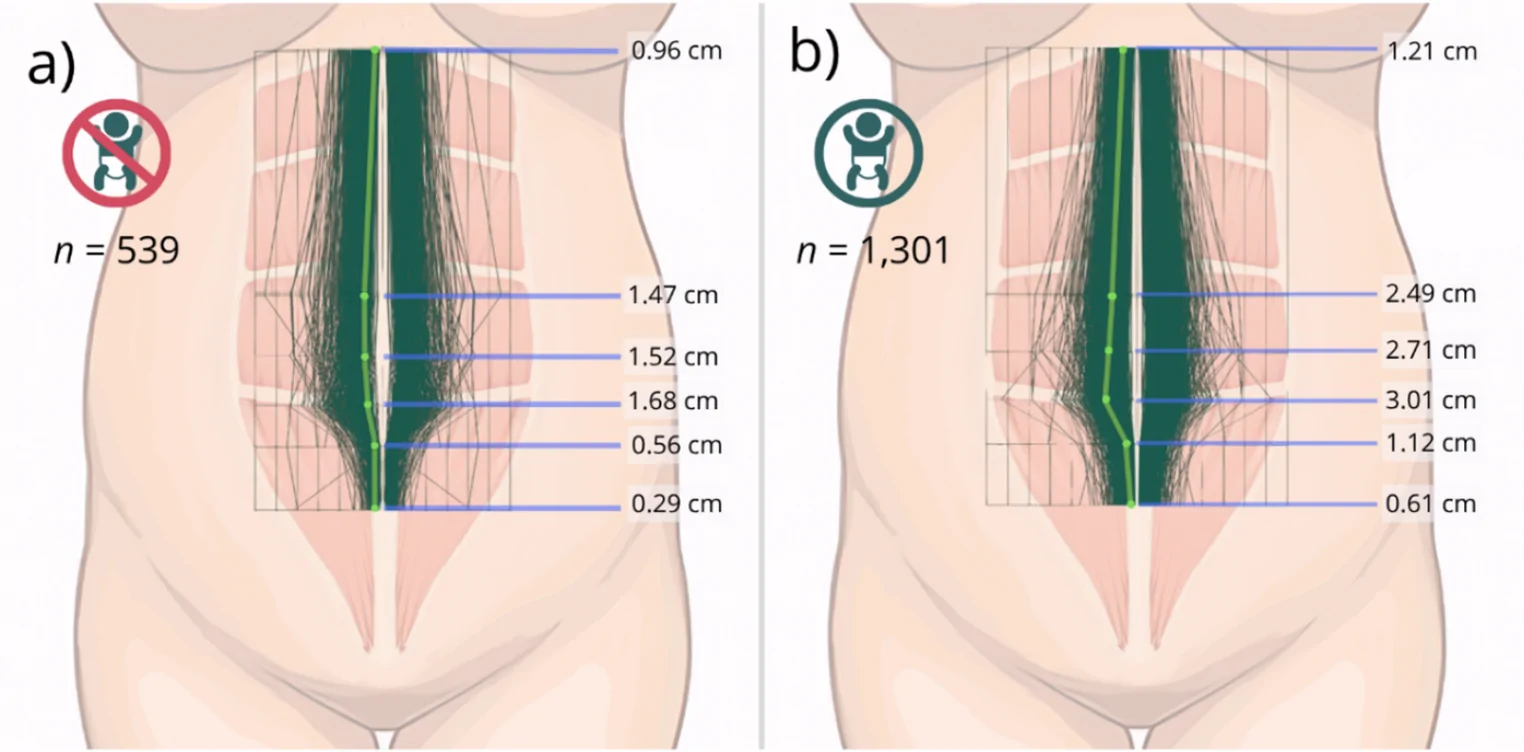
Figure illustrates the variation in the inter-rectus distance (IRD) among two groups: (**a**) nulliparous females and (**b**) parous females. The dark green, fine lines depict the measurement results for each study participant, collectively representing the diverse manifestations of IRD. The light green single thick lines with dots represent reported mean values of the IRD, as well as the mean measures in centimeters (cm)

**Fig. 4:**
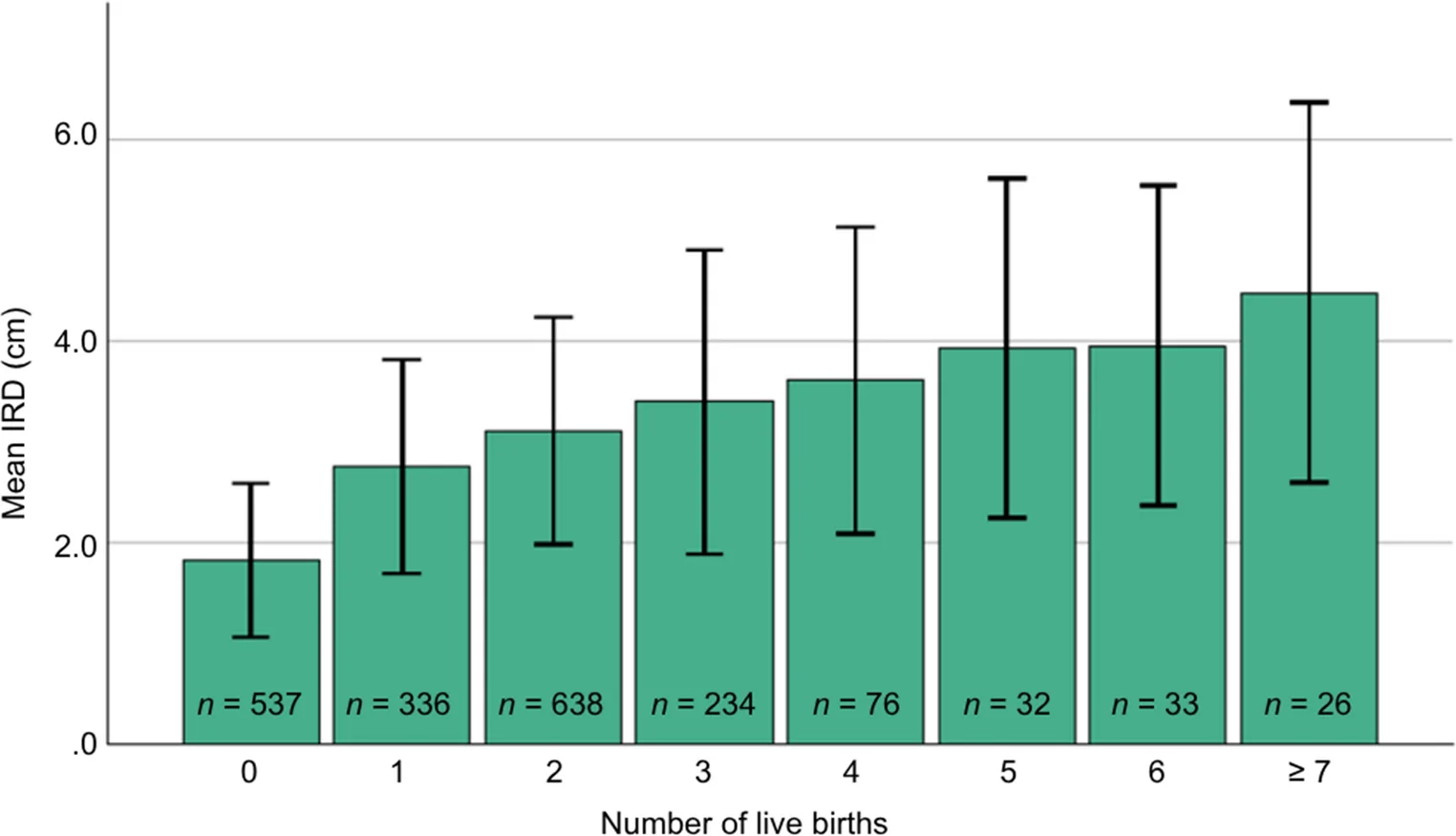
Parity across 8 categories as a function of inter-rectus distance (IRD) is depicted, with bars representing the mean IRD and error bars indicating ± 1 standard deviation

When stratified by parity, clear differences in the IRD distribution were observed. Among the nulliparous participants (*n* = 539), the mean IRD measured 3 cm above the umbilicus was 1.52 cm (SD 0.70) ranging from 0.35 to 4.37 cm. In contrast, parous participants (*n* = 1,301) exhibited substantially wider separation, with a mean IRD of 2.71 cm (SD 1.18), ranging from 0.46 to 9.86 cm. The distributions of IRD at this measurement site for nulliparous and parous participants are illustrated in Fig. 3. Figure 4 further illustrates parity across the eight categories by showing the mean IRD values, highlighting the distance variability within each category.

**Table 2:** Inter-rectus distance (IRD) measures from anatomical points and maximal IRD from any location. Values are presented separately for nulliparous and parous participants, as well as for the total study population. Measurements are shown as mean (cm), also standard deviation. Sample sizes (*n*) vary by measurement site due to differences in measurement availability across anatomical points

	Total			Non-parous			Parous		
	*n*	Mean	Std. deviation	*n*	Mean	Std. deviation	*n*	Mean	Std. deviation
Xiphoideum	1837	1.14	0,60	520	0.97	0.53	1317	1.21	0.62
4.5 cm from xiphoideum	1327	2.23	1.16	334	1.47	0.76	993	2.49	1.16
3 cm above umbilicus	1835	2.38	1.19	520	1.52	0.70	1315	2.71	1.17
Umbilicus	1320	2.68	1.26	333	1.68	0.76	987	3.01	1.21
2 cm below umbilicus	1836	0.96	0.80	520	0.56	0.44	1316	1.12	0.86
4.5 cm below umbilicus	1327	0.53	0.57	334	0.29	0.36	993	0.61	0.61
Maximal IRD, cm	1837	2.77	1.30	520	1.81	0.75	1317	3.16	1.27

### Comparison with previously published reference values

To facilitate comparison with previously published population-based reference values, IRD was additionally examined using the percentile-based approach proposed by Kaufmann et al. [13]. Applying a cutoff corresponding to their suggested upper limit of “normal” (IRD ≥ 3.4 cm at 3 cm above the umbilicus), 16.5% (*n* = 315) of participants in the present cohort were classified above this threshold. In the current dataset, the 80th percentile of the IRD measured at the same anatomical location was 3.24 cm.

### Distribution of maximal IRD relative to commonly used cutoff values

The distribution of IRD was examined in relation to two commonly applied cutoff values, 3 cm and 5 cm, using the maximal IRD measured at any measuring point.

In the entire analytical sample, 38.5% of participants (*n* = 737) exhibited a maximal IRD of ≥ 3 cm, whereas 5.4% (*n* = 99) exceeded the 5 cm cutoff value. Among nulliparous participants, 93.7% exhibited a maximal IRD of < 3 cm, and 99.8% had a maximal IRD of < 5 cm. In contrast, among parous participants, 49.6% had a maximal IRD of < 3 cm, while 92.5% had a maximal IRD of < 5 cm. Maximal IRD values ranged from 0.35 to 9.86 cm.

When examining the population distribution of maximal IRD, from any measuring point, percentile analyses demonstrated that the 80th percentile corresponded to 3.76 cm, the 90th percentile to 4.39 cm, and the 95th percentile to 5.09 cm.

### Location of maximal IRD along the linea alba

**Fig. 5:**
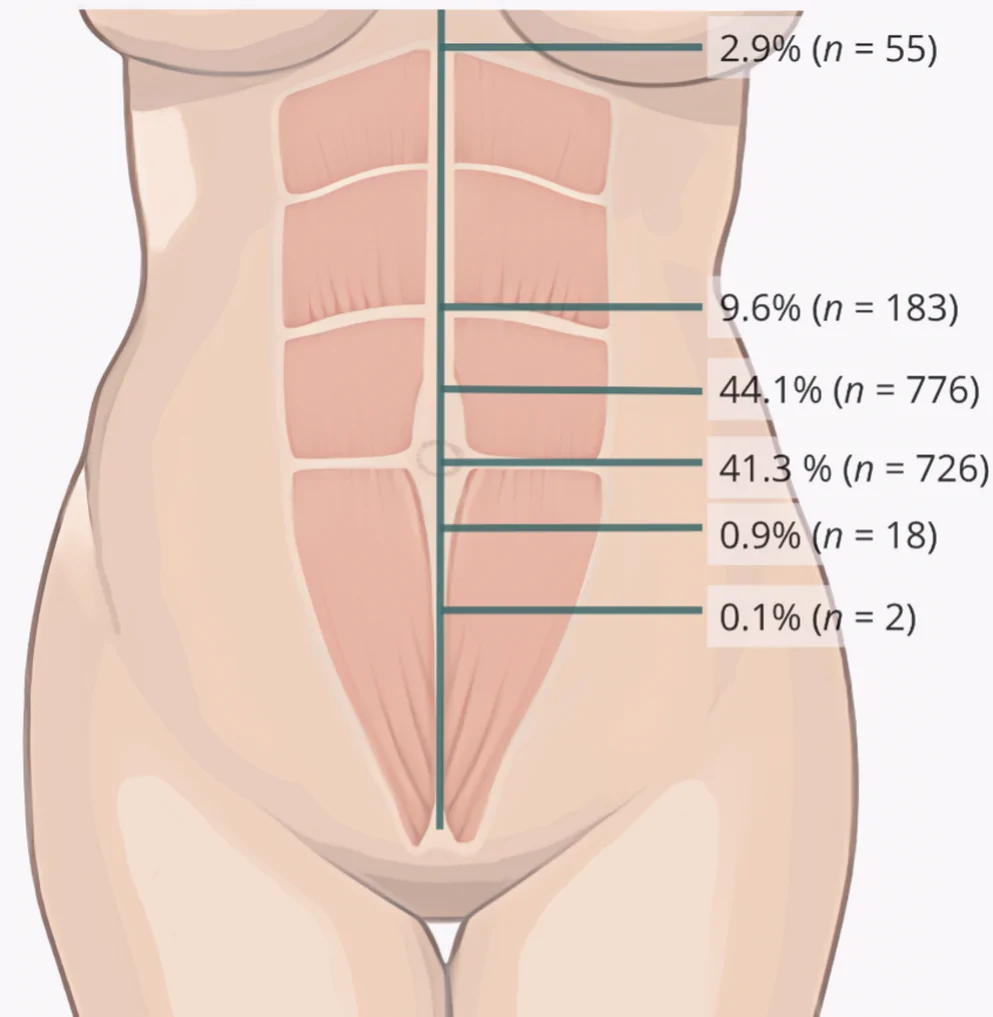
The distribution of maximal inter-rectus distance measurement points, expressed as percentages and numbers of examinees (*n*)

Among participants in whom the maximal IRD was observed at one of the six predefined measuring points (*n* = 1,760), the maximal separation was most frequently located at the umbilicus (41.3%, *n* = 726) or 3 cm above (44.1%, *n* = 776), while an additional 9.6% of maximal measurements were located 4.5 cm above the umbilicus (*n* = 183) (Fig. 5). In contrast, maximal IRD was infrequently observed below the umbilicus, with 0.9% (*n* = 18) located 2 cm below and 0.1% (*n* = 2) located 4.5 cm below the umbilicus (*n* = 2). In 2.9% of participants (*n* = 55), the maximal IRD was located at the xiphoid level. In addition, for 81 participants (4.3%), the maximal IRD was located outside the predefined measuring points and could therefore not be assigned to a specific standard measurement location. The distribution of maximal IRD locations along the linea alba is illustrated in Fig. 5.

### Associations between maximal IRD and anthropometric variables

Maximal IRD, defined as the largest separation observed at any predefined anatomical measurement site, showed weak positive correlations with BMI (ρ = 0.227), waist circumference (ρ = 0.273) and body weight (ρ = 0.199) (all *p* < 0.001), whereas no meaningful correlation was observed with height (ρ = −0.049, *p* = 0.034). When stratified by parity, the association between maximal IRD and BMI was stronger among nulliparous participants (ρ = 0.338, *p* < 0.001) than among parous participants (ρ = 0.228, *p* > 0.001).

In linear regression analyses restricted to participants with naturally occurring IRD, parity was strongly associated with the maximal IRD. Each additional childbirth was associated with a 0.42 cm increase in maximal IRD (B = 0.417, 95% CI 0.384–0.451, *p* < 0.001). BMI was also independently associated with maximal IRD, although with a smaller effect size (B = 0.061 cm per kg/m², 95% CI 0.052–0.070, *p* < 0.001).

## Discussion

This population-based study represents one of the largest clinical analysis of IRD to date, measuring IRD at multiple anatomical sites and defining the maximal IRD per individual to capture the natural linea alba variation. Over one-third of women exceeded the ≥ 3.0 cm IRD threshold, and 16.5% surpassed ≥ 3.4 cm. Percentile analysis indicated that these thresholds lie within the population reference distribution, with values near 5 cm representing the upper tail (95th percentile). Parity showed a strong independent association with maximal IRD, whereas BMI had a weaker but statistically significant effect on IRD. Maximal IRD was most frequently observed at or above the umbilicus. Awareness of DRA was quite common among examinees, as 75% had heard of DRA.

Previous IRD research has predominantly focused on pregnant or postpartum women or specific subgroups, limiting insights into anatomical variations in the general population [6, 8, 9]. For example, Beer et al. (2009) measured IRD in nulliparous women (*n* = 150) excluding parous subjects and those with higher BMI, restricting generalizability of their findings. In contrast, our study’s representative cohort with minimal exclusion criteria captures the heterogeneity of parity and body size, thereby providing a more comprehensive description of the IRD variation across adult women. The observed right-skewed IRD distribution aligns with imaging and cadaveric studies demonstrating variability in linea alba morphology [2, 15]. This broader sampling enhances the understanding of IRD as a continuous trait rather than a dichotomous condition.

IRD exhibits a continuous distribution without a discrete biological cutoff, consistent with imaging and cadaveric evidence showing gradual linea alba width variation, being widest above the umbilicus and narrowing caudally [1, 2]. Our findings support the notion that IRD reflects connective tissue morphology rather than pathology. Dichotomizing IRD using fixed thresholds obscures its continuous nature and inflates the prevalence estimates of significant IRD values [6]. Maintaining IRD as a continuous variable preserves anatomical variation and avoids information loss that is inherent in categorical classification. The maximal inter-rectus distance (IRD) was most consistently measured at or above the umbilicus, consistent with findings from previous studies [2, 8, 15].

There is uncertainty about how fixed numerical cutoffs relate to population reference. Percentile-based reference values are reflecting anatomical variations and enable cross-study comparisons. Barbosa et al. (2013) reported a mean supraumbilical linea alba width of ~ 2.2 cm in cadavers without defects, supporting distribution-based limits [21]. Kaufmann et al. (2022) observed that over half their cohort exceeded a 2 cm cutoff at 3 cm supraumbilical, while an IRD of ~ 5 cm corresponded to the 95th percentile, which is consistent with our findings [13]. Similarly, Tuominen et al. (2022) showed that DRA prevalence depends heavily on the cutoff choice, with severe widening (> 5 cm) being uncommon [22]. High-resolution morphometric analyses confirm continuous linea alba width variation peaking near the umbilicus, supporting percentile-based interpretation over fixed thresholds [3, 15]. Cavalli et al. (2021) demonstrated that prevalence estimates vary sharply with threshold selection, reinforcing the limitations of fixed cutoffs [6]. It is emphasized that cutoffs are not inherently wrong or automatically lead to oversteer.

In this study, the upper limit of IRD is established as the 95th percentile of the examinees. This decision is based on three factors. First, there is no externally validated biological threshold for pathological IRD; studies report cutoff values ranging from 15 mm to over 34 mm without reaching a consensus, therefore, interpreting IRD relative to its population distribution is the most defensible approach [12, 13]. Secondly, the IRD distribution is right-skewed, making parametric methods inappropriate; the 95th percentile does not rely on any assumptions about the distribution shape and is defined as the value below which 95% of observations fall [23]. Lastly, because only unusually large values are of interest, we utilized a one‑sided upper reference limit at the 95th percentile. This approach allows for 5% of an otherwise unselected population to fall above the limit [24]. Descriptive limits of the IDR in the 80th or 90th percentile are generally inconsistent with standard reference interval practices, if employed without adequate methodological justification [24]. The use of the 95th percentile aligns with anthropometric conventions aimed at identifying rare variants rather than redefining “normal” anatomy.

Parity emerged as a dominant independent predictor of maximal IRD, while BMI exerted a weaker, context-dependent influence. Wu et al. (2021) linked obesity to increased DRA prevalence in older women, possibly due to elevated intra-abdominal pressure, whereas Qu et al. (2021) found weak correlations between BMI and IRD in early postpartum women, diminishing with higher parity [25, 26]. These findings corroborate our results, indicating pregnancies substantially shapes population-level IRD variation rather than BMI in reproductive aged women.

### Strengths, limitations, and future directions

Strengths of our study include its large, population-based design and the use of standardized ultrasound measurements at multiple anatomical sites performed by trained clinicians, which creates clearer picture of linea alba heterogeneity and maximal value of IRD along the continuum of the midline.

Limitations include homogeneity in age and ethnicity, which restricts generalizability to diverse populations and men. In addition, the lack of detailed postpartum timing data prevents stratified analyses. The exclusion of women within six months postpartum mitigates early recovery bias. While the findings describe anatomical variation robustly, we must emphasize that they do not establish clinical significance until the symptoms and risk factors are further investigated. The current data provides a reference distribution, not a diagnostic criteria.

Future research should integrate anatomical data with functional effects and patient-reported symptoms to delineate clinically meaningful linea alba widening. Longitudinal studies encompassing broader age ranges, male subjects, and diverse ethnicities are essential to refine reference values and informed diagnostic criteria of DRA.

## Conclusion

Population‑based ultrasound data show that IRD exhibits substantial normal anatomical variation in adult women. Fixed IRD cutoffs commonly used to define DRA fall within the population distribution, with values up to approximately 5 cm corresponding to the 95th percentile. These findings support interpreting IRD as a continuous anatomical characteristic rather than a dichotomous pathological entity.

## Data Availability

The datasets used and analyzed are available from the University of Oulu, https://www.oulu.fi/en/university/faculties-and-units/faculty-medicine/northern-finland-birth-cohorts-and-arctic-biobank/womens-health-study

## Footnotes

1 The term “Caucasian” reflects the original survey wording. It is recognized as an imprecise and outdated descriptor and should not be interpreted as equivalent to White, Western European or European race, ethnicity, nationality, or genetic ancestry.
